# The relationship between SGLT2 and systemic blood pressure regulation

**DOI:** 10.1038/s41440-024-01723-6

**Published:** 2024-05-23

**Authors:** Priscilla Ahwin, Diana Martinez

**Affiliations:** Department of Biomedical Sciences, Cooper Medical School of Rowan University, 401 South Broadway, Camden, NJ 08103 USA

**Keywords:** Hypertension, Neural control, Nucleus of solitary tract, Sodium-glucose cotransporter 2, Sodium-glucose cotransporter 2 inhibitors

## Abstract

The sodium-glucose cotransporter 2 (SGLT2) is a glucose transporter that is located within the proximal tubule of the kidney’s nephrons. While it is typically associated with the kidney, it was later identified in various areas of the central nervous system, including areas modulating cardiorespiratory regulation like blood pressure. In the kidney, SGLT2 functions by reabsorbing glucose from the nephron’s tubule into the bloodstream. SGLT2 inhibitors are medications that hinder the function of SGLT2, thus preventing the absorption of glucose and allowing for its excretion through the urine. While SGLT2 inhibitors are not the first-line choice, they are given in conjunction with other pharmaceutical interventions to manage hyperglycemia in individuals with diabetes mellitus. SGLT2 inhibitors also have a surprising secondary effect of decreasing blood pressure independent of blood glucose levels. The implication of SGLT2 inhibitors in lowering blood pressure and its presence in the central nervous system brings to question the role of SGLT2 in the brain. Here, we evaluate and review the function of SGLT2, SGLT2 inhibitors, their role in blood pressure control, the future of SGLT2 inhibitors as antihypertensive agents, and the possible mechanisms of SGLT2 blood pressure control in the central nervous system.

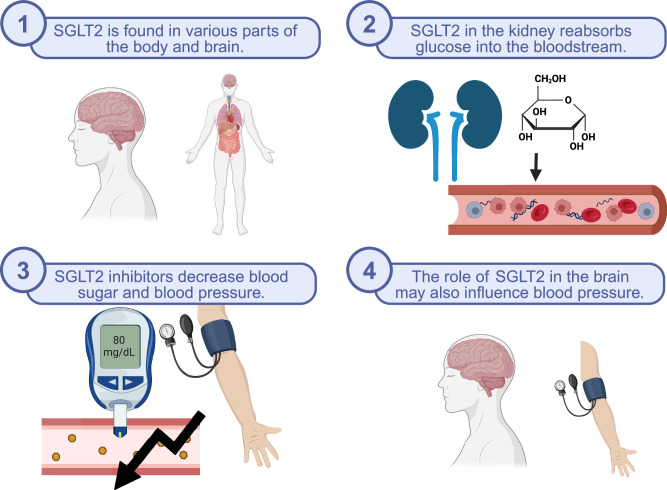

## Introduction

**Fig. 1:**
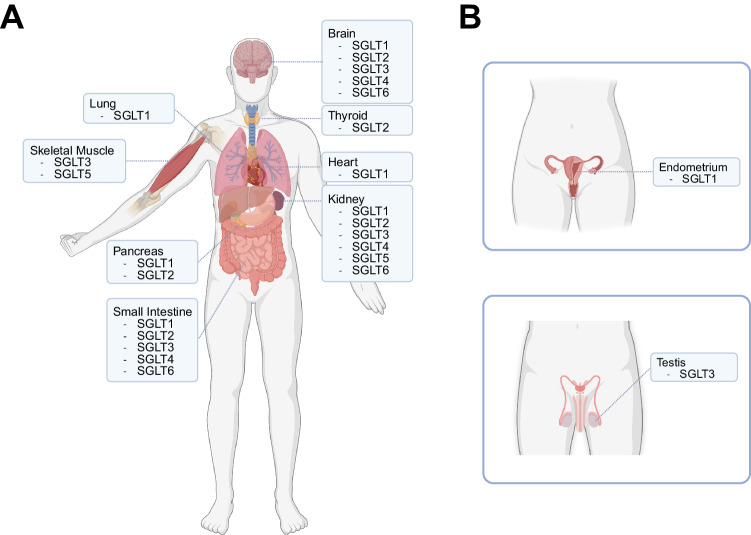
Sodium-glucose-linked transporters (SGLTs) currently known locations. **A** SGLTs are widely distributed throughout the human body, residing in various organs. **B** SGLTs are differentially found in the reproductive systems, SGLT1 in the endometrium and SGLT3 in the testis

Sodium-glucose cotransporter 2 (SGLT2) is part of a class of transporters called sodium-glucose-linked transporters (SGLTs) [1]. Prior to the 1960s, scientists understood that glucose could pass through the brush border of the small intestine through active transport [1]. The mechanism by which this took place was not completely understood until Bob Crane hypothesized a model for active transport in 1960 [1–4]. Crane suggested that the energy needed for active transport was dependent on the sodium-potassium pump, a protein found in the cell membrane [2, 5]. The sodium-potassium pump works by creating a gradient of sodium ions [1]. SGLTs undergo an active transport process by obtaining energy from this gradient. Although this concept was not initially received, Peter Mitchell took Crane’s concept and conceived the word *symport* to describe secondary active transport [6]. SGLTs are symporters that pass both glucose and sodium, Na^+^, across the cell membrane, a process that aids in regulating blood glucose levels [1]. Later evidence would then support Crane’s theory, and SGLT protein and activity were eventually discovered in numerous parts of the body, including the skeletal muscle, heart, and lung (Table 1, Fig. 1) [7–10].

**Table 1:** SGLT isoform localization

Location	SGLT isoform	Literature source
Brain	SGLT1	[69]
	SGLT2	[60]
	SGLT3	[70]
	SGLT4	[71]^b^
	SGLT6	[70, 72]
Salivary glands	SGLT2	[71]^b^
Thyroid	SGLT2	[73]^a^
Skeletal muscle	SGLT3	[7, 8]
	SGLT5	[7]
Lung	SGLT1	[9]
Heart	SGLT1	[10]
Liver	SGLT1	[74]
	SGLT4	[71]^b^
	SGLT5	[7]
Pancreas	SGLT1	[75, 76]
	SGLT2	[77]^a^
Kidney	SGLT1	[17]
	SGLT2	[78]
	SGLT3	[79]
	SGLT4	[80]
	SGLT5	[7]
	SGLT6	[72]
Small intestine	SGLT1	[81, 82]
	SGLT3	[8]
	SGLT4	[80]
	SGLT6	[72, 83]
Endometrium	SGLT1	[84]
Testis	SGLT3	[71]^b^

Sodium-glucose linked transporters (SGLTs) isoforms and location in different tissue types. SGLTs are a class of transporters with numerous isoforms. Isoforms are widely distributed throughout the body

^a^In some tissues, activity of SGLT2 inhibitors was present

^b^Secondary literature supports the presence of these isoform locations

It would take approximately 20 years after Crane’s hypothesis to identify SGLT1 by using photoaffinity labeling, amongst other techniques [11, 12]. Concurrently, research indicated the presence of high-affinity and low-affinity transporters [13]. Stoichiometry demonstrated that the high-affinity transporters had a sodium-to-sugar ratio of 1 to 1 while low-affinity transporters had a sodium-to-sugar ratio of 2 to 1 [13]. The difference in SGLT affinities was differentiated as SGLT1 and SGLT2. SGLT1 was later cloned, the chromosomal location discovered, and the human gene family SLC5, which includes SGLTs, was eventually established [14].

**Fig. 2:**
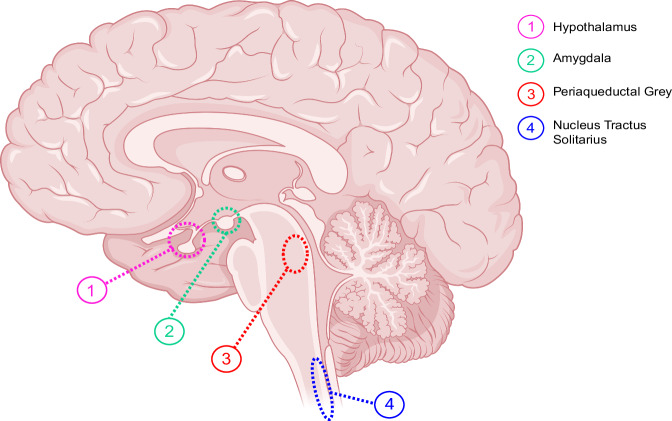
Hemisection of the brain and the distribution of SGLT2. SGLT2 resides in the Hypothalamus (1), Amygdala (2), Periaqueductal Grey (3), and Nucleus Tractus Solitarius (4)

SGLT2 is found in the proximal convoluted tubule of the nephron [15]. S1 and S2 segments of the nephron express SGLT2 [16]. The receptor resides on the apical surface of the proximal tubule’s epithelial cells, while GLUT2 resides on the basolateral surface [16]. When the body begins to generate filtrate that will eventually become excretable urine, the role of SGLT2 in the nephron is to reabsorb glucose from the filtrate. More than 90% of the glucose reabsorbed from the filtrate is accounted for by SGLT2, while the other 10% is due to SGLT1 [15, 17, 18]. Besides this, SGLT2 also has an impact on blood pressure, as SGLT2 inhibitors (SGLT2i) not only have glycemic control but also a secondary effect on blood pressure. Furthermore, SGLT2 is not only found in the kidney, but also in the central nervous system (Fig. 2) [19, 20]. SGLT2 receptors may use central nervous system mechanisms to provide cardioprotective effects by influencing areas involved in cardiorespiratory regulation.

This review explores the importance of the SGLT2 receptor in blood glucose and blood pressure regulation. It also examines the clinical significance SGLT2 inhibitors have on blood pressure in diabetic and nondiabetic individuals. In addition, this review discusses the SGLT2 localization in different cardiorespiratory centers, as well as the current studies examining its connection to the cardiorespiratory functions of brainstem nuclei. SGLT2 inhibitors have displayed clinical application, and understanding their effects on the brain can broaden their use in medicine.

## SGLT2 and diabetes

While there are up to six SGLT isoforms, SGLT2 has recently been an appealing transporter to target due to its significance in diabetes mellitus type 2 [21, 22]. Diabetes mellitus (DM) is a chronic metabolic disease that causes high blood glucose levels as a result of insulin deficiency or insulin resistance. Insulin is a peptide hormone created by the beta cells of the pancreas [23]. When food is consumed, it is broken down into smaller molecules, including glucose. Glucose serves as a vital energy source in the nervous system, creates macromolecules, and performs various metabolic processes [24]. Because of its various functions, glucose needs to be transported from the blood to the inside of cells to aid in glycolysis and oxidative phosphorylation, metabolic processes that produce adenosine triphosphate (ATP), a molecule that provides energy for the cells. Insulin is released from the beta cells of the pancreas when food is consumed and allows for glucose uptake into cells [23]. Insulin binds to insulin receptors on cell membranes, which then triggers the activation of glucose transporters [23]. Once glucose transporters are activated, glucose can enter a cell and perform its functions.

Diabetes mellitus comes in different forms  including type 1 and type 2. Type 1 diabetes mellitus (T1DM) is an autoimmune disorder that results in the destruction of insulin-producing pancreatic beta cells [23]. Type 2 diabetes mellitus (T2DM) occurs when the pancreas does not release as much insulin as needed or when cells have decreased sensitivity to insulin. In T2DM, it is recommended that hemoglobin A1c is less than 7% [25]. Hemoglobin A1c is a way of quantifying blood sugar levels by measuring the degree of glycosylated sugar in the blood for an average of three months [26, 27].

**Fig. 3:**
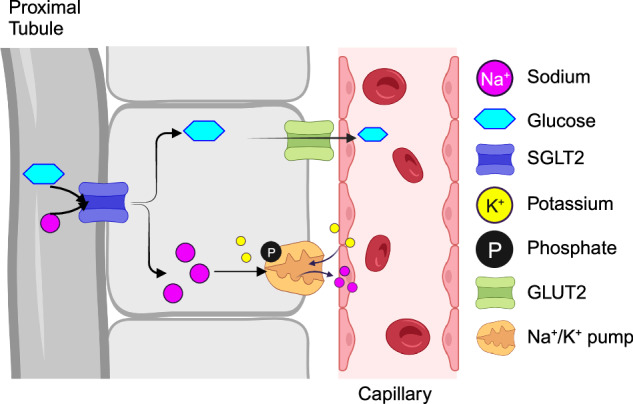
Glucose reabsorption at the proximal convoluted tubule (PCT) through SGLT2. Under normal physiological conditions, the kidney reabsorbs most of the body’s filtered glucose. This occurs via SGLT2 in the early proximal tubules, and SGLT1 in the more distal regions of the PCT. Sodium (pink) and glucose (aqua) travel together from the apical side of the PCT. Once in the cell of the PCT, sodium and glucose travel to the bloodstream (capillary) separately using the Na/K^+^ pump (orange) and GLUT2 (green), respectively

One of the biggest threats that diabetic patients face is hyperglycemia and its serious consequences. To mitigate this problem, researchers found an advantage in targeting SGLT2 [1]. The kidneys are an important regulator of blood sugar homeostasis, and the process is highly dependent on SGLT2, a key protein on the apical membrane of the proximal tubule of the nephron [16]. As waste material begins to be generated in the kidney, it is co-mingled with beneficial substances, such as glucose. The kidney is responsible for filtering the glucose back into the body while excreting the waste material as urine [16]. SGLT2 works through a two-stage process, in which glucose and sodium go through the SGLT2 transporter into the cell body (Fig. 3) [16]. Accumulation of glucose in the cell causes its exit to the plasma through the glucose transporter 2 (GLUT2), and the Na^+^/K^+^ pump works to maintain sodium concentration by pumping sodium into the plasma [16]. In a nonhuman primate model, tofogliflozin and phlorizin, competitive inhibitors of SGLT2, caused the excretion of glucose through the urine, leading to numerous discoveries exploring the impact of SGLT2 inhibitors [28].

**Fig. 4:**
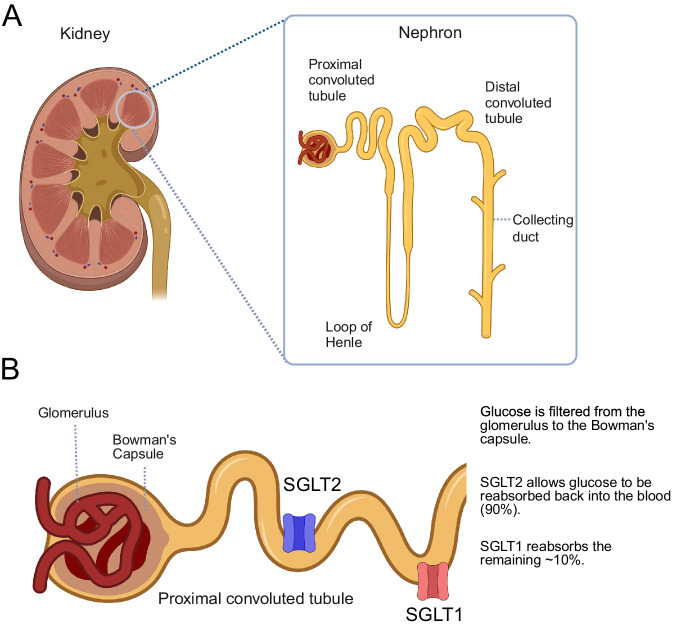
SGLT2 action in the kidneys. **A** The nephron is composed of the glomerulus, proximal convoluted tubule (PCT), loop of Henle, distal convoluted tubule, and collecting duct. Glucose is filtered from the glomerular capillary into Bowman’s capsule. **B** Glucose travels through the PCT and is reabsorbed into the bloodstream by SGLT2 (blue) and SGLT1 (red). When SGLT2 is inhibited, approximately 90% of filtered glucose cannot be reabsorbed and is eventually excreted as part of urine

SGLT2i are a recent class of antidiabetic medication that cause glucosuria by inhibiting glucose absorption in the proximal tubule of the nephron [29, 30]. When an inhibitor against SGLT2 is introduced, SGLT1-mediated transport increases as a compensatory measure in glucosuria. Still, inhibiting SGLT2 mitigates hyperglycemia and increases urine glucose [17] (Fig. 4). Instead, it travels through the nephron, is excreted through the urine, and subsequently decreases blood sugar. Recent research revealed that in addition to reducing blood sugar, SGLT2i also decreases blood pressure in diabetic hypertensive individuals [31]. The mechanism by which blood pressure is decreased is not entirely understood, but it is possible that osmotic and natriuretic diuresis decreases circulating plasma volume eventually leading to decreased blood pressure [32, 33]. Additionally, SGLT2 may influence the sympathetic nervous system.

In mice and healthy humans, the SGLT2i empagliflozin treatment resulted in urinary glucose excretion. A short-term experiment by Lin et al. showed that during a 7-day treatment of empagliflozin, blood glucose levels exhibited a continuous decrease in diabetic mice [34]. The long-term experiment after 10 weeks of treatment revealed that mice with obesity and type 2 diabetes had a significant improvement in cardiovascular abnormalities and cognitive function [34]. A study by Seman et al. demonstrated that empagliflozin caused dose-dependent glycosuria in healthy male individuals without inducing hypoglycemia [35]. Similarly, a Phase I trial of empagliflozin showed that a dose increase resulted in a greater cumulative glucose excretion [36]. A single dose of empagliflozin can result in urinary glucose excretion of 46.3 to 89.8 g over 24 hours as opposed to 5.84 g with placebo [37]. Ultimately, empagliflozin treatment stimulated excretion of urinary glucose, subsequently reducing blood glucose acutely and HbA1c chronically [38]. This is consistent with data from SGLT2 knockout mice showing similar results [38].

## SGLT2 inhibitors have a secondary effect on Blood Pressure

High blood pressure, or hypertension, is an exceedingly prevalent public health condition affecting one third to almost half of all adults in many countries including the United States, Japan, Argentina, Paraguay, Uruguay, Canada, and Germany [39–43]. Hypertension is defined by the American Heart Association as: Stage 1 systolic between 130–139 mmHg or a diastolic between 80–89 mmHg, and Stage 2 systolic as 140 mmHg or higher or a diastolic between 90 mmHg or higher. The World Health Organization (WHO) defines high blood pressure as a systolic greater than 140 mmHg or diastolic greater than 90 mmHg. By the year 2025, it is predicted that 1.56 billion adults worldwide will die due to hypertensive complications [16, 44].

Hypertension is linked to a greater risk of cardiovascular disease, and it is also a major risk factor for all-cause morbidity and mortality [45]. Studies show that individuals develop hypertension due to an interplay of factors that involves an individual’s genetic makeup and environmental influences [45]. Individuals with hypertension can successfully lower their blood pressure by increasing physical activity and modifying dietary needs [45]. While lifestyle changes are encouraged, some individuals will rely on antihypertensive medications, many of which work by utilizing the body’s natural pathways to regulate high blood pressure. For example, medications like angiotensin-converting enzyme (ACE) inhibitors influence the renin–angiotensin–aldosterone system (RAAS) pathway, subsequently preventing an increase in sympathetic activity and hindering the release of the hormone aldosterone [46]. A cumulation of these effects makes ACE inhibitors a reliable medication against hypertension. While first line antihypertensives like ACE inhibitors have been used clinically for decades, advances in antihypertensive medications show a promising future for sodium-glucose cotransporter-2 (SGLT2) inhibitors. SGLT2 inhibitors utilize various mechanisms to decrease systolic and diastolic blood pressure while also reducing the risk of heart failure and cardiovascular death [47].

Nguyen et al. explored the effects of dapagliflozin, an SGLT2i. Mice were split into two groups and given either a dose of the inhibitor by an intragastric gavage method of the SGLT2i or an analogous dose of saline [48]. Two hours after administration, cardiovascular parameters, including blood pressure, were measured. Results of the study revealed that the control’s systolic blood pressure after two hours was greater compared to the experimental blood pressure. The same was true of diastolic blood pressure. Both results revealed that the inhibitor decreased blood pressure significantly (*P* < 0.05) [48]. The study by Nguyen et al. demonstrates how blood pressure is impacted by SGLT2i. Nguyen et al. noted that seizure activities were inhibited by dapagliflozin, suggesting the role of SGLT2 in neural electrophysiology [48]. Immunohistochemical tests later confirmed SGLT2 residing in brain regions responsible for autonomic regulation [48]. The conclusion of the study affirmed that inhibition of SGLT2 by dapagliflozin impacts central autonomic control [48]. It is possible that the reason SGLT2i can regulate blood pressure is because the sympathetic process in the central autonomic system is inhibited by SGLT2i, as SGLT2 is distributed in brain areas specific to autonomic control.

Tikkanen et al. showed that the SGLT2i empagliflozin is effective in patients with comorbid diabetes and hypertension [49]. The study recruited patients with both hypertension and type 2 diabetes and gave one group either empagliflozin or a placebo for 12 weeks. 10 mg of empagliflozin reduced blood pressure by 3.44 mmHg while 25 mg of empagliflozin decreased blood pressure by 4.16 mmHg [49]. In line with this study, Ferdinand et al. conducted a study in which patients were assigned to an empagliflozin or placebo group [50]. At week 24 of the experiment, there was a significant reduction in 24 hour ambulatory systolic blood pressure in patients receiving empagliflozin. Additionally, the effect was comparable to conventional antihypertensive monotherapies [50]. Empagliflozin has a clinically relevant reduction in blood pressure [51, 52]. The cardiorenal effects of SGLT2i differ depending on race. Notably, a study by Kunutsor et al. showed that Asian and White patients with T2DM taking SGLT2i have a decreased risk of major adverse cardiovascular events and a reduced risk of nephropathy [53]. Regional differences on the effectivity of SGLT2i, however, were not observed [53].

An investigation from Kim et al. reveals that SGLT2i has a role in non-diabetic models. The findings from this study showed that in prehypertensive rats, ongoing administration of SGLT2i reduces heart rate and resting blood pressure while attenuating the progression of hypertension [54]. Kim et al. divided young spontaneously hypertensive rats (SHRs) into two groups, one as the control group and one that would be administered the SGLT2i dapagliflozin. Beginning at 4 weeks of age, both groups were fed their respective diets with the treatment group receiving a dapagliflozin-containing diet. By 8 weeks of age, the dapagliflozin-administered group had reduced daytime/nighttime mean arterial pressure compared to the control group. A study by Kravtsova et al. supports the above findings. Non-diabetic Dahl salt-sensitive (Dahl SS) rats were divided into two groups, one as a control group and one given dapagliflozin [55]. Administration of dapagliflozin reduced salt-induced hypertension in the rats while increasing glucose and sodium excretion. Using this Dahl SS study, researchers concluded that a relationship exists between SGLT2 inhibition, blood pressure, and the renin-angiotensin-aldosterone system (RAAS). Treatment with dapagliflozin dampens salt-induced hypertension and does not influence kidney injury. These two studies contribute to the idea that SGLT2 inhibitors can benefit patients with hypertension regardless of diabetic status [55].

Human studies also show that SGLT2 inhibitors have a positive effect on nondiabetic patients [56]. A systemic review and meta-analysis showed that compared to patients not taking SGLT2 inhibitors, patients without diabetes mellitus taking SGLT2 inhibitors have a statistically significant reduction in body weight, systolic blood pressure, and fasting plasma glucose [56]. One study included in the article by Teo et al. highlighted that cardiovascular death or aggravated heart failure was decreased in the treatment group receiving empagliflozin [57]. Additionally, another study revealed that canagliflozin significantly decreased body weight in overweight and obese subjects without diabetes mellitus compared to the placebo group [58]. These results illustrating the benefits of SGLT2i in nondiabetic individuals are consistent with a double-blind, randomized, placebo-controlled clinical trial using dapagliflozin [59]. The trial was performed on 50 patients with prediabetes and prehypertension [59]. Patients taking 10 mg of dapagliflozin once daily for 90 days exhibited a reduction in 24-hour and nighttime systolic blood pressure, ultimately decreasing blood pressure variability [59].

## SGLT2 in the central nervous system

Chiba et al. found that SGLT2 is generally expressed in human and rat brains [60]. This was done using immunohistochemistry on autopsies of human brains and C3H/He mouse brains. The results showed that cells of the choroid plexus were positive for the expression of SGLT2/SLC5A2 [60]. The presence of SGLT2 in the brain highlights its function beyond the kidney. For example, findings from a study conducted by Oerter et al. presented that SGLT2 may be used as a biomarker of traumatic brain injury [61]. The study suggested that SGLT2 expression was significantly upregulated following trauma to the cerebral hemisphere after 72 hours, indicating a relationship between SGLT2 protein expression and survival time after a traumatic brain injury [61]. Further SGLT2 functions can be explored to investigate the use of SGLT2 inhibitors.

The distribution of SGLT2 in the brain extends from the forebrain (telencephalon and diencephalon) to the midbrain and brainstem [48]. Oshima et al., showed SGLT2 and SGLT1 within rostral ventrolateral medulla (RVLM) [32]. The RVLM in the brainstem triggers neurogenic hypertension by inciting sympathetic nerve activity [62]. The goal of the study was to determine if bulbospinal neurons, which supplies information to preganglionic neurons of the sympathetic nervous systems [62], respond to SGLT2 and SGLT1 inhibitors and thus alleviate hypertension by weakening activity in the sympathetic nervous system [63]. Histological analysis was employed to affirm the presence of SGLT2 and SGLT1 receptors residing in RVLM neurons [63]. Additionally, bulbospinal cells hyperpolarized when exposed to SGLT2 and SGLT1 inhibitors [63]. The results concluded that combined therapy of SGLT2 and SGLT1 inhibitors may decrease blood pressure by influencing activity of neurons in the RVLM through suppression of neuronal activity [63]. SGLT2 is found in many different regions within the brain including the nTS. C-Fos is a protein whose expression indicates neuronal activity. Table 2 shows that within the brainstem, the density of SGLT2 and c-Fos expression is greatest in the nTS [48]. Research also reveals that mice under dapagliflozin treatment expressed greater c-Fos immunoreactivity in the nTS compared to controls [48]. Quantitative measures denoted a statistically significant difference, with the dapagliflozin-treated group having Fos-immunoreactivity of 95.500 ± 3.704 compared to the control group, which was 50.000 ± 3.661. Literature indicates that SGLT2 receptors are present in the brain, especially in the nucleus tractus solitarius (nTS). The nTS resides in the medulla oblongata, where it is the first synaptic station for the body’s cardiorespiratory afferent inputs [64, 65]. It receives signals from chemoreceptors, baroreceptors, and cardiopulmonary receptors [64, 66, 67]. If the nTS receives a signal that the blood pressure is too high or too low, it will send signals to other areas of the brain that result in changes to heart rate and vessel width to help the body regulate optimal blood pressure. The exact mechanism by which the nTS regulates blood pressure has not been fully elucidated. One mechanism may be through the action of excitatory and inhibitory neurotransmitters, as well as astrocytes. Another mechanism may be through catecholaminergic neurons in the nTS [68]. It is possible that SGLT2 may also play a role in regulating blood pressure in the nTS, albeit through a different mechanism than that of the kidney. Understanding the function and mechanism of how SGLT2 functions in the nTS will help reveal how the nTS regulates blood pressure. This can aid in unique treatment developments that target areas in the brain responsible for blood pressure regulation. SGLT2 may influence cardiovascular functions in the nTS, and studies suggest that SGLT2 inhibitors may have a role to play in the brain aside from their well-known antihyperglycemic effects [60]. Furthermore, if SGLT2 has a similar mechanism in the brain as in the kidney, one would expect that having fewer SGLT2 receptors would result in decreased blood pressure compared to having more SGLT2 receptors. Thus, individuals with higher blood pressure may either have increased activity of SGLT2 or be predisposed to possess more SGLT2 receptors, causing elevated blood pressure.

**Table 2:** SGLT2 density and c-Fos expression based on brainstem location

Brainstem location	SGLT2 density	c-Fos expression
Parabrachial nucleus	Moderate	Moderate
Stratum griseum centrale	Moderate	Moderate
Locus coeruleus	Low	Moderate
Lateral reticular nucleus	Moderate	Moderate
Nucleus of solitary tract	High	High
Area postrema	Moderate	Low

SGLT2 density and c-Fos expression in different areas of the brainstem. However, expression for both is highest in the nTS. Table adapted from [48]

## Conclusion

The body is able to regulate processes like blood sugar and blood pressure levels in remarkably unique ways. This review explored how the SGLT2 receptor functions in the body to maintain blood glucose levels, notably by reabsorption of glucose across the proximal convoluted tubule. The emergence of SGLT2 inhibitors demonstrates that SGLT2 not only regulates blood sugar levels, but also has the capacity to influence blood pressure through postulated mechanisms such as osmotic diuresis, natriuresis, and modulation of the sympathetic nervous system. The regulation of blood pressure is one that can be used to address hypertension in populations where its prevalence is increasing dramatically. This dual role of SGLT2 is partially fascinating given its presence in the nTS, which regulates cardiorespiratory functions. The nTS may be influenced by the expression of SGLT2. Current experiments show that the expression of SGLT2 is neuroprotective. Further research must be carried out to understand how the presentation of SGLT2 in the nTS specifically changes in hypertensive compared to normotensive individuals. Furthermore, the mechanisms that SGLT2 uses in the nTS to affect blood pressure must be investigated. With this information, researchers can seek therapeutic treatments that lower blood pressure by targeting the nTS directly through SGLT2 inhibitors.
